# Comparison of Algorithms for Prediction of Protein Structural Features from Evolutionary Data

**DOI:** 10.1371/journal.pone.0150769

**Published:** 2016-03-10

**Authors:** Robert P. Bywater

**Affiliations:** Francis Crick Institute, London NW7 1AA, England; University of Queensland, AUSTRALIA

## Abstract

Proteins have many functions and predicting these is still one of the major challenges in theoretical biophysics and bioinformatics. Foremost amongst these functions is the need to fold correctly thereby allowing the other genetically dictated tasks that the protein has to carry out to proceed efficiently. In this work, some earlier algorithms for predicting protein domain folds are revisited and they are compared with more recently developed methods. In dealing with intractable problems such as fold prediction, when different algorithms show convergence onto the same result there is every reason to take all algorithms into account such that a consensus result can be arrived at. In this work it is shown that the application of different algorithms in protein structure prediction leads to results that do not converge as such but rather they collude in a striking and useful way that has never been considered before.

## Introduction

Although protein structure determination by biophysical techniques such as X-ray crystallography, cryoelectron microscopy and NMR has become highly automated, there will, for several reasons, continue to be interest in pursuing theoretical predictions of protein structure. Despite the high productivity of the mentioned experimental methods, the rate at which genomics and proteomics data are generated still outstrips the rate at which structures can be determined experimentally. Performing mutant studies for planning protein engineering experiments or screening for proteomic therapeutics (for example: immunotherapeutics) are most rapidly done *in silico*. Further, it is not simply the case that any given gene has a (singular) function. The protein prescribed by its gene sequence has many functions [1,2]. This implies, in turn, that these are also encoded in the gene. Somewhere, but where and how? As earlier shown [1,2] this is done in a disjoint fashion. So the problem becomes an issue of how to partition the protein sequence information and map these subsets of the entire gene sequence onto this set of functions (the inherent assumption in all this, which dealt with in more detail elsewhere, is that the mapping of sequence *loci* into function space is both surjective and injective). While many of these issues have been addressed in recent [1] and earlier [2] publications the focus here is on folding and contact prediction and not on any of the other functions that any given protein most certainly has.

Theoretical/computational protein folding studies have undergone a steady series of developments in recent years. These have included significant accomplishments in protein dynamics [3–5], methods based on a collage of overlapping peptide fragments [6], and a variety bioinformatics approaches [7–12]. The latter have usually involved finding patterns of coevolution within multiple sequence alignments. The so-called correlated mutation analysis (CMA) approach identifies residue positions that show a common pattern of conservation and are deemed to signify the maintenance of some key structural feature, a “contact”. Typically, what one had in mind in these studies was protein folding, the need for the protein to fold into domains with a compact (predominantly “hydrophobic”) core. A similar argument was used to propose that protein-protein interactions could likewise be predicted [13,14].

As an extension of the CMA idea, studies of patterns of sequence variability (VAR) and Shannon entropy (ENT) [15,16] allowed a distinction to be made between sites in the protein core, or surrounding ligand-binding sites, for example. The first steps towards unravelling the multifunctional nature of proteins [1,2] had been taken. This was recently supplemented by an alternative approach based on Kolmogorov complexity (KOL) [1] which represents a new way to partition protein sequence information into its constituent functionalities.

In this paper, the focus will be restricted to protein folding, or more specifically, the folding of individual domains. The extent to which KOL, and its antecedents [15,16] VAR/ENT (here considered jointly and called VRN), can be used to predict these domain structures will be considered, as well as alternative methods. Foremost among these are methods which have been based on frequency of contacts between amino acid residue sidechains [17,18]. In the present work distinction is made between an earlier method [17] in which a PDB-derived likelihood matrix was used to predict intradomain contacts (referred to herein as the SVB method) and a later development based on pair-to-pair contacts [18] (P2P). The P2PConPred program [18] calculates correlations between sites based on a predefined P2P matrix which in turn is based on the Blocks database [19]. The P2P website states: “The P2P is currently designed to reflect probabilities of pair to pair substitutions at two positions with physical contact. The ultimate goal is to detect residue-residue contact solely based on the evolutionary information stored in multiple sequence alignment.”. The present paper includes results from the use of the P2P program but proceeds towards the same ultimate goal in ways that P2P probably did not envisage.

## Methods

**Fig 1:**
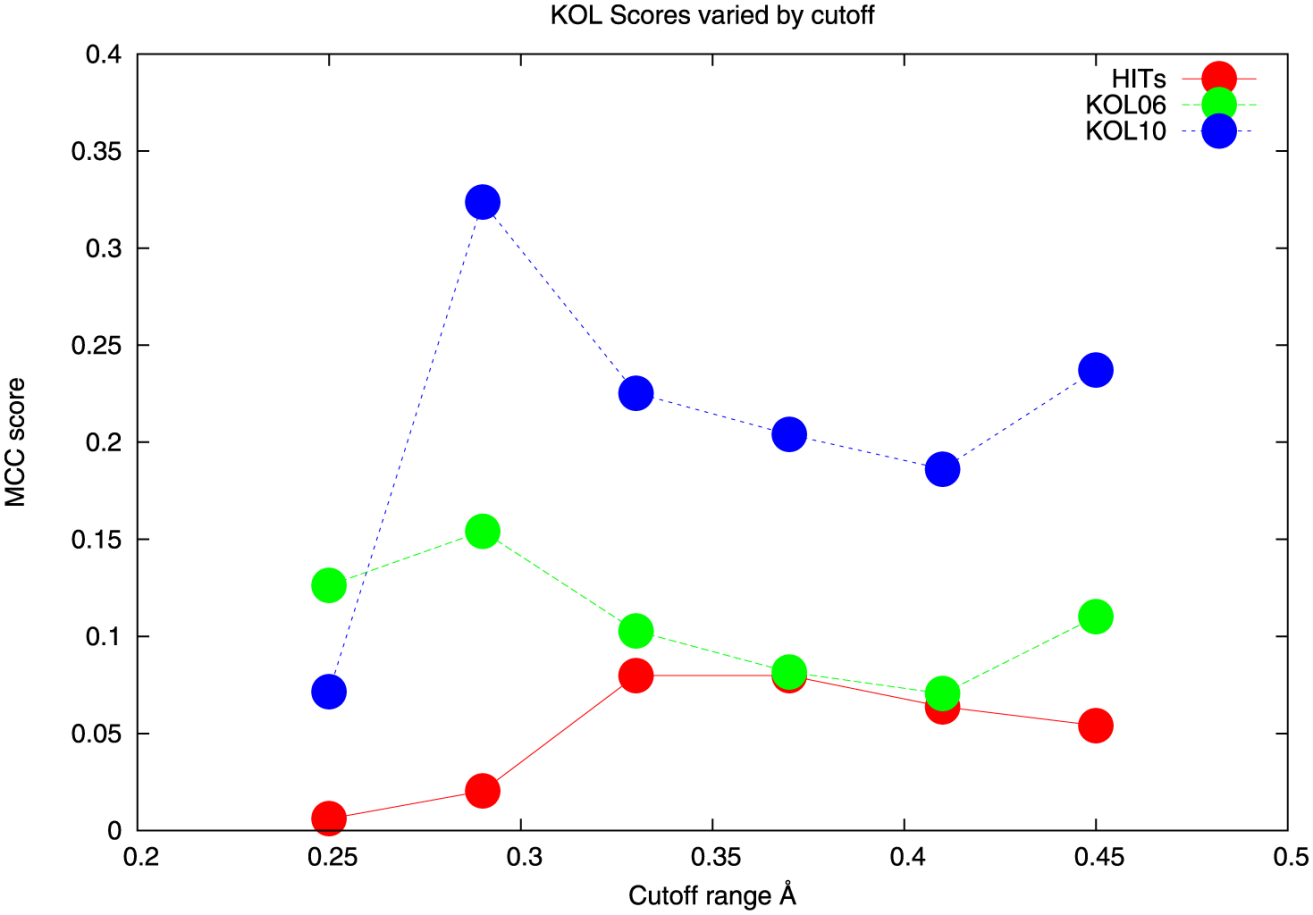
Selection of cutoff ranges for KOL. The abscissa of each data point indicates the center of the range, all ranges have a width of ± 0.05 on the KOL scale. The measurements were made at cutoff distances 6Å (KOL06) and 10Å (KOL10). The total number of hits is shown in red.

Before the VRN and KOL measurements can be made it is important to decide the range of values that give results that are relevant to the type of contact being studied. This question has been studied earlier for VRN [15,16] and KOL [1]. For VRN, the values obtained in the original work [15,16] and used elsewhere [1] were used. In the case of KOL the results of making these investigations were not published before so this is done here. Reference is made to Fig 1 which shows how the MCC values for KOL calculated at two different distance cutoffs, 6Å and 10Å (these turn out to be good choices as the later results show, but other values could have been chosen) vary as a function of the range of KOL values is varied, in the range 0.2 to 0.5, the width of each slice of that range is 0.05. As is shown in Fig 1, the optimal MCC value is the same for 6Å and 10Å (and intervening values, data not shown).

**Fig 2:**
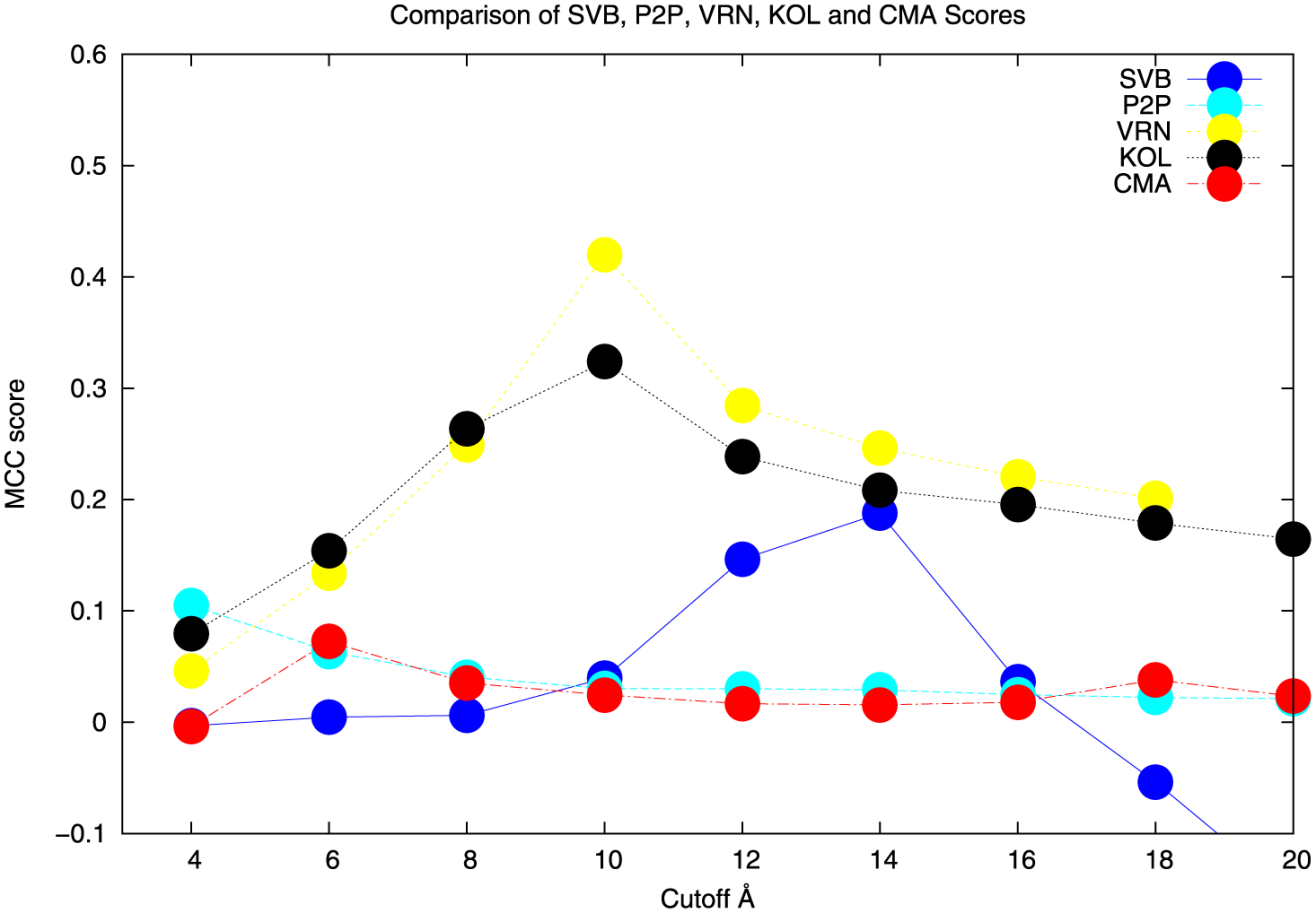
Contact distance plots for the nonredundant set of 10 proteins for CMA, KOL, VRN, P2P and SVB (text colours here correspond to the colours in the figures). The identities of the proteins for each plot are listed in Table 2 - column 1: PDB I.d., column 2: working name for the protein, column3: figure number.

The data to produce Fig 2 and S1 Fig and the Tables 1 and 2 come from the following sources:

- VRN and KOL: were determined using previously published methods [1] for dealing with a specially designed nonredundant database of protein domains. Briefly, for each protein a multiple sequence alignment was produced using the PredictProtein program [20]. This program generates VAR and ENT data, although these need to be parsed and extracted into a usable form. The KOL data are not provided directly but can be calculated based on the complexity of the alignments at each position in the consensus sequence. These methods are all described in detail in [1].
- SVB: previously published [17] contact matrix.
- P2P: http://ignmtest.ccbb.pitt.edu/p2pdocs/p2p_doc.html
- CMA: http://gremlin.bakerlab.org/sub.php

**Table 1:** Full results for all methods for the protein 1a4v (item 1 in Table 2).

Algorithm	Cutoff Å	TP	FP	FN	TN	MCC	ACCY	PREC	SENSY	MCC corr
**SVB**	4	7	54	938	6504	-.0031	0.866	0.115	0.007	0.0032
	6	22	156	837	6488	0.0045	0.862	0.124	0.026	0.0248
	8	34	279	713	6477	0.0063	0.859	0.109	0.046	0.0441
	10	62	494	498	6449	0.0397	0.851	0.112	0.111	0.1073
	12	107	796	197	6403	0.1463	0.839	0.118	0.352	0.2589
	14	146	1123	130	6104	0.1876	0.794	0.115	0.529	0.3583
	16	198	1514	521	5270	0.0366	0.676	0.116	0.275	0.2831
	18	256	1870	877	4500	-.0537	0.566	0.120	0.226	0.2715
	20	310	2253	1260	3680	-.1564	0.449	0.121	0.197	0.2673
**P2P**	4	7	61	23	7412	0.1499	0.987	0.103	0.233	0.1050
	6	7	162	78	7256	0.0432	0.966	0.041	0.082	0.0635
	8	9	286	202	7006	0.0029	0.933	0.031	0.043	0.0407
	10	13	501	417	6572	-.0374	0.874	0.025	0.030	0.0302
	12	23	802	718	5960	-.0835	0.791	0.028	0.031	0.0301
	14	32	1130	1046	5295	-.1417	0.701	0.028	0.030	0.0290
	16	38	1520	1436	4509	-.2217	0.596	0.024	0.026	0.0248
	18	43	1876	1792	3792	-.3030	0.500	0.022	0.023	0.0222
	20	47	2260	2176	3020	-.4026	0.396	0.020	0.021	0.0211
**KOL**	4	13	47	331	7112	0.0733	0.946	0.217	0.038	0.0796
	6	35	148	229	7091	0.1339	0.940	0.191	0.133	0.1542
	8	58	272	106	7067	0.2258	0.934	0.176	0.354	0.2636
	10	102	487	109	6805	0.2561	0.893	0.173	0.483	0.3237
	12	153	788	410	6152	0.1259	0.800	0.163	0.272	0.2385
	14	200	1116	738	5449	0.0376	0.700	0.152	0.213	0.2083
	16	266	1506	1128	4603	-.0510	0.578	0.150	0.191	0.1955
	18	304	1862	1484	3853	-.1465	0.473	0.140	0.170	0.1787
	20	340	2246	1868	3049	-.2591	0.361	0.131	0.154	0.1646
**VRN**	4	10	61	374	7058	0.0398	0.939	0.141	0.026	0.0461
	6	35	162	272	7034	0.1134	0.933	0.178	0.114	0.1337
	8	66	286	149	7002	0.2112	0.924	0.188	0.307	0.2490
	10	120	501	66	6816	0.3254	0.892	0.193	0.645	0.4200
	12	187	802	367	6147	0.1717	0.794	0.189	0.338	0.2843
	14	245	1130	695	5433	0.0757	0.691	0.178	0.261	0.2464
	16	306	1520	1085	4592	-.0260	0.571	0.168	0.220	0.2205
	18	350	1876	1441	3836	-.1241	0.465	0.157	0.195	0.2011
	20	387	2260	1825	3031	-.2406	0.352	0.146	0.175	0.1921
**CMA**	4	0	61	88	7354	-.0099	0.980	0.000	0.000	-0.0036
	6	3	162	13	7325	0.0522	0.976	0.018	0.188	0.0725
	8	5	286	137	7075	-.0026	0.942	0.017	0.035	0.0352
	10	7	501	352	6643	-.0430	0.884	0.014	0.019	0.0246
	12	8	802	653	6040	-.0960	0.804	0.010	0.012	0.0166
	14	9	1130	981	5383	-.1551	0.716	0.008	0.009	0.0156
	16	15	1520	1371	4597	-.2286	0.611	0.010	0.011	0.0179
	18	24	1876	1727	3876	-.3039	0.513	0.013	0.014	0.0382
	20	28	2260	2111	3104	-.4003	0.410	0.012	0.013	0.0234

In this table the following statistical checks were carried out, in accordance with recently established practice [1,21]:

MCC (Matthew's Correlation Coefficient):

(TP*TN–FP*FN)/√((TP+FP)*(TP+FN)*(TN+FP)*(TN+FN))

Accuracy (ACCY): (TN–TP)/(TP+FP+FN+TN)

Precision (PREC): TP/(TP+FP)

Sensitivity (SENSY): TP/(TP+FN)

**Table 2:** Summary of results for all 10 protein families (parent protein identified in columns 1 and 2).

Protein domain (4-letter code)	Protein type	Figure (A-I in S1 Fig)	CATH class (click on links for details including 3D structure)	Peaks in contact distances vs MCC plots (secondary peaks)				
				CMA	KOL	VRN	P2P	SVB
1a4v_	α-lactalbumin	2	1.10.530.10	6.0	(8.0) 10.0	8.0 12.0	~	14.0
5tim_	TIM barrel	A	3.20.20.70	5.0 (8.0)	6.0 (5.0)	10.0 (8.0)	12.0 (10.0)	~
1ewka	receptor ligand binding domain	B	3.40.50.2300	-5.5 (4.0)	6.0	-4.5 & -5.0 (5.5)	10.0	~
1fw0a	receptor membrane domain	C	3.40.190.10	5.5	5.5	5.5	~	~
1ulkb	lectin	D	3.30.60.10	5.5	4.0 (6.0)	5.5	6.0	10.0
1kx5e	histone	E	1.10.20.10	5.5	5.0	8.0	12.0	4.5
2b4sb	insulin receptor TK domain	F	2.60.40.1410	8.0 (5.0)	8.0	10.0	14.0	~
1xcka	GroEL	G	3.30.260.10	8.0 (6.0)	8.0	8.0	12.0	~
1bpya	DNA β polymerase	H	3.30.210.10	8.0 (5.5)	3.8 (5.5)	8.0	(5.0)	~
1n8ka	alcohol dehydrogenase	I	3.40.50.720	8.0 (5.0 & 6.0)	6.0 (5.0)	8.0	8.0	~

For all of the above methods, comparisons were made between “hits” identified by the method and those in the 2D contact map for each target protein (listed in Table 2, columns 1 & 2). Contact maps at cutoff values 3.8, 4.0, 4.5, 5.0, 5.5, 6.0, 8,0, 10,0, 12.0, 14,0, 16.0, 18,0 and 20.0 were calculated and used for these comparisons. A count was made of true and false positives to calculate the Matthews correlation coefficients (MCCs) ([1,21] See also caption to Table 1) for each method at each cutoff value. The MCCs are plotted along the abscissae and the cutoffs form the ordinates of the plots in Fig 2 and S1 Fig.

The results of applying these methods to the target proteins in this work are shown in Fig 2 and S1 Fig. Table 1 records the data for a single member of the set of proteins PDB i.d.:1a4v. (Corresponding data for all the others is available from the author) and Table 2. The members of the studied protein set (Table 2 columns 1 & 2) were chosen according to dual requirements for wide coverage of domain fold space (Table 2 column 4) and accuracy of the crystal structures (R and B-values obtainable through the links in column 4 of Table 2). Structural data including rotatable figures are also reachable through the same links.

The question of noise and random effects in all this data has not been ignored; quite the converse. For each of the above metrics, the behaviour of a set of predictions based on completely random inputs was calculated and used to correct the metrics (subtraction of RND).

**Fig 3:**
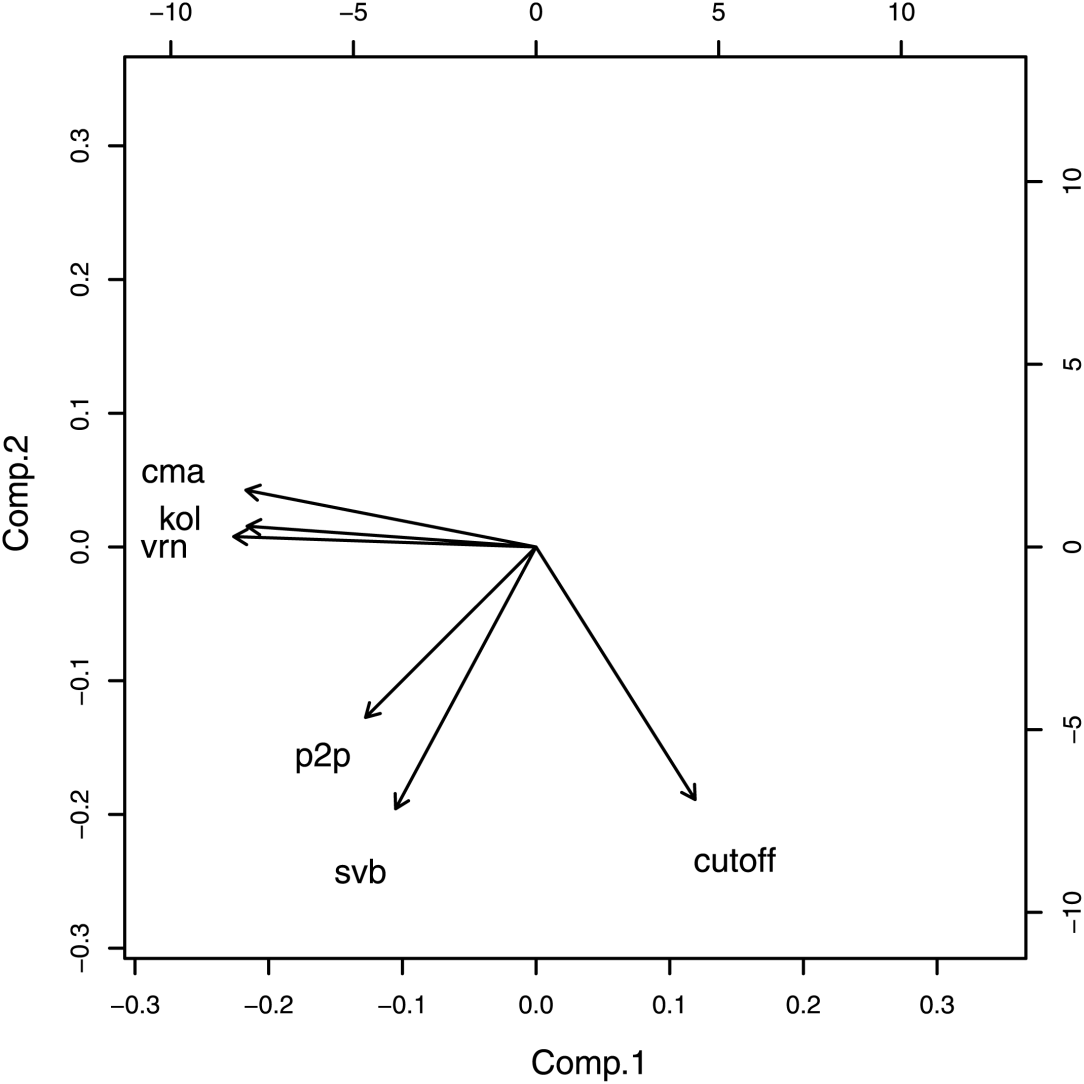
Principle component analysis of CMA, KOL, VRN, P2P and SVB for the entire set of protein domains.

Statistical correlations between CMA, KOL, VRN, P2P and SVB (corrected for RND) displayed (Fig 3) as a principle component analysis diagram. The first two components ((dominant–see insert) are plotted. This diagram was produced using the statistics package R (http://www.r-project.org/).

## Results and Discussion

The data arising out of this work can be used to construct 2D contact maps from which 3D structures can, in principle, always be derived using distance geometry [22–26]. There are ample precedents for presenting folding predictions in this way [1,10,11,17]. But now there is a new and better definition of “contact” because now we can define contacts in terms of preferred rather than just assumed (see below) distances, depending on the method used (Table 1). In addition to this enhanced contact data there is a wealth of other data which have earlier been enlisted in these endeavours such as predictions of secondary structure [1,10] and accessibility [1] which confer additional credence to the results of any attempts to compute the 3D structure.

Once these juxtaposed results have been presented, the next step is to decide how best to combine the 2D contact predictions from SVB, P2P, VRN, KOL and CMA in such a way as to provide the best consensus set, “best” being here defined as leading to rapid convergence towards a structure for the protein that corresponds to the crystal structure for this protein. As stated, there has been widespread interest in trying to predict intra-protein (as well as inter-protein) contacts. But very little is ever said about the nature of those contacts. The definition of “contact” can be very vague or ambiguous, often referring to “hydrogen bond” or “Van der Waals” contacts. Neither of these include the possibility of and maybe even need for long range effects that are not contacts as such. A standard (http://www.ccp4.ac.uk/html/contact.html) range for short contacts is 2.0Å-3.66Å while a considerably wider range, 6Å-12Å, is considered significant in order to cater for all contact types (http://en.wikipedia.org/wiki/Protein_contact_map). Given that there is such a wide spread of distances which are involved in defining a “contact” it now becomes interesting and, as it turns out, important to ask the question: in each of the algorithms for contact prediction listed above and summarised in Table 1, what is the characteristic contact distance for each of these algorithms? The answer is provided by the cutoff distance for each case where MCC is a maximum. When this is done it becomes apparent that the various algorithms predict contacts having different characteristic distances. A clear conclusion from this work is that there is no “one-size-fits-all” algorithm for inter-residue contact prediction. One would clearly not choose the SVB or P2P alternatives since their behaviour is somewhat erratic and often confined to predicting rather long spacings (>10Å). There might be correlated behaviour over such long distances, but they can hardly be considered a “contact”. But it obviously makes sense to use CMA, VRN and KOL.

One may legitimately ask why, given that CMA got off to such a good start in fold prediction, is there any need to consider other methods? Do the apparent correlations in CMA really correspond to events in coevolution? There have been many discussions on this question [12,27,28] and more remains to be discovered. In particular, there are clear indications [28] that CMA “hits” may reflect the rate of coevolution in relation to preserving arenaceous (i.e. low resolution) structural features such as the protein “core” rather than acting as a predictor of specific pairs of contacting residues as such. But insofar that CMA can with appropriate noise filters be used to predict contacts the Gremlin approach [29] is most useful and it produces results of very high fidelity.

This paper has its main focus on protein folding, or rather, domain folding. Several different methods for predicting domain folds were compared and it was found that these methods work in subtly different ways in that they predict contacts with different values. There is every reason therefore to use more than one, but not all, of these methods. Together they provide a more robust and information-rich prediction model and, while they do not “converge” as such, they “collude” in a way that could to lead to a more reliable result (at least as far as VRN and KOL are concerned).

From Fig 3 it would appear that KOL, VRN and CMA are controlled by similar underlying factors and all three correlate in an almost antiparallel fashion with cutoff. Of course, there is no linear correlation as is made abundantly clear in Fig 2 and S1 Fig. P2P and SVB are almost orthogonal to the cutoff indicating little or no dependency in that sense.

One of the missing items in much published work is a clear definition of what is meant by “contact”. A mention of this has been made [28] which amounts to a general assumption throughout the CMA debate that, for example a “hydrophobic-hydrophobic” contact can be replaced by a hydrogen bond or a salt-bridge or an “aromatic-aromatic” contact. As if these were freely interchangeable. But they are not interchangeable in such a simple way [30]. These interactions are based on entirely different mechanisms and replacement of one by the other is not to be regarded as a “compensatory mutation” [30]. Another missing item in previously published work is that there has been such a focus on “contacts”, however these are defined and/or measured, that other most important protein functions seem to have been forgotten. Exceptions to this is a precursor to the present paper [1] and a most important earlier paper [31] that sets out to consider the ability to disentangle direct and indirect correlations and to facilitate computational predictions of alternative protein conformations, protein complex formation, and even the *de novo* prediction of protein domain structures. Together with the efforts of the present author, this seems to be a valid and useful way forward. To this end, future extensions of this work will give further consideration to these other protein functions [1] that are encoded in the gene (the ability to fold into two (or more) conformational states, to be able to reach one state from the other, arriving at the correct locus inside or outside the cell, or within the cell membrane, recognition/binding to other proteins, recognition/binding of small ligands (orthosteric and/or allosteric agents)). Indeed, much of the difficulty surrounding the use of these contact prediction methods arises out of the fact that so many different functions are encoded in the gene and attempts to partition them lead to the kind of results that have been revealed in this work. Thus the use of the verb “disentangle” [31] is highly appropriate in this context.

## Conclusions

This paper has dealt with the question of which inputs to use when conducting *ab initio* predictions of domain folds. Five methods were compared and it was found that they all make predictions in different ways. Different in respect to which interatomic distance or displacement that they best predict. CMA, VRN and KOL emerge as being the most useful methods for predicting “contacts”. The first two are already well established, while the Kolmogorov approach [1, 32] represents a novel and promising addition to the arsenal of techniques.

As for the interatomic contacts themselves, no account has been taken here of the nature of the atom types involved (but it already is one of the ongoing extensions from this work). Here, a standard “CA-CA” proximity metric is assumed as a definition for all “contacts”. But the issue is an important one. Depending on the chemical nature of the participating atom types the problem (generally) of finding matching pairs amounts is a case of the mathematically well defined “marriage problem” (Gale-Shapley algorithm). This is applicable to “+/- type” interactions or wherever there is a duality or asymmetry in the interaction. But there are also interactions of a more neutral or symmetric kind such as “hydrophobic” interactions typical of the way that aliphatic, and to some extent aromatic, sidechains interact. These have more the character of the “stable-roommate problem” (Irving algorithm). It is intended that these distinctions will form the basis of yet another extension of this work.

## Supporting Information

S1 Fig This file contains figures A, B, C, D, E, F, G, H and I. The identities of the proteins is stated in columns 1 and 2 of Table 2. Software developed for this paper is all available free of charge to interested users. The source code for the fortran program (*predcon*.*f*) which calculates the MCC scores from the input data is provided and users are strongly advised to peruse this file before use making note of the comments and the default names of the input files. Examples of the input files are provided for the purposes of getting the correct format. The program should be run once for each cutoff value and it is advisable to rename the output files using a filename that incorporates that value A shellscript (*renumber*) is provided to help with that and an awkscript (*awkward*) is provided to enable the different measurements (CMA, VRN, etc.) to be corrected for the random (RND) scores. The correct command for compilation of predcon.f under Linux is:
*gfortran -Wall -O3 -ffixed-line-length-132 -o predcon*.*exe predcon*.*f*
(GZ) Click here for additional data file.

S1 Software The software is available in S1 Software. It goes without saying that no commercial use may be made of these programs or scripts in whole or in part without express permission of the author.
(GZ) Click here for additional data file.

## References

[1] Bywater RP (2015) Prediction of protein structural features from sequence data based on Shannon entropy and Kolmogorov complexity. PLoS ONE April 9, 1–15 (10.1371/journal.pone.0119306) PMC4391790 25856073

[2] Bywater RP (2013) Protein folding: a problem with multiple solutions. J Biomol Struct Dyn 31: 351–362. (10.1080/07391102.2012.703062) 22870987

[3] Duan Y, Kollman PA. (1998) Pathways to a protein folding intermediate observed in a 1-microsecond simulation in aqueous solution. Science 282: 740–744. 10.1126/science.282.5389.740 9784131

[4] Shaw DE, Maragakis P, Lindorff-Larsen K, Piana S, Dror RO, Eastwood MP, et al (2010) Atomic-level characterization of the structural dynamics of proteins. Science 330: 341–346. (10.1126/science.1187409) 20947758

[5] Seddon GM, Bywater RP (2012) Accelerated simulation of unfolding and refolding of a large single chain globular protein. Open Biol 2: 120087 (10.1098/rsob.120087) 22870389 PMC3411113

[6] Simons KT, Kooperberg C, Huang E, Baker D (1997) Assembly of protein tertiary structures from fragments with similar local sequences using simulated annealing and Bayesian scoring functions. J Mol Biol 268: 209–225. (10.1006/jmbi.1997.0959) 9149153

[7] Altschuh D, Lesk AM, Bloomer AC, Klug A (1984) Correlation of coordinated amino acid substitutions with function in viruses related to tobacco mosaic virus. J Mol Biol 193: 693–707. (10.1016/0022-2836(87)90352-4) 3612789

[8] Göbel U, Sander C, Schneider R, Valencia A (1994) Correlated mutations and residue contacts in proteins. Proteins 18: 309–317. (10.1002/prot.340180402) 8208723

[9] Marks DS, Hopf TA, Sander C (2012) Protein structure prediction from sequence variation. Nature Biotechnol 30: 1072–1081. 23138306 10.1038/nbt.2419 PMC4319528

[10] Taylor WR, Jones DT, Sadowski MI (2012) Protein topology from predicted residue contacts. Protein Sci 21: 299–305. (10.1002/pro.2002) 22102360 PMC3324774

[11] Taylor WR, Hamilton RS, Sadowski MI (2013) Prediction of contacts from correlated sequence substitutions. Curr Opinion Struct Biol 23: 473–479. (10.1016/j.sbi.2013.04.001) 23680395

[12] Jones DT, Buchan DWA, Cozzeto D, Pontil M (2012) PSICOV: precise structural contact prediction using sparse inverse covariance estimation on large multiple sequence alignments. Bioinformatics 28: 184–190. (10.1093/bioinformatics/btr638) 22101153

[13] Pazos F, Helmer-Citterich M, Auselio G, Valencia A (1997) Correlated mutations contain information about protein-protein interaction. J Mol Biol 271: 511–523. (10.1006/jmbi.1997.1198) 9281423

[14] Ovchinnikov S, Kamisetty H, Baker D (2014) Robust and accurate prediction of residue–residue interactions across protein interfaces using evolutionary information. ELife 3: e02030 (10.7554/eLife.02030.001) 24842992 PMC4034769

[15] Oliveira L, Paiva PB, Paiva AC, Vriend G (2003) Identification of functionally conserved residues with the use of entropy-variability plots. Proteins 52: 544–552. (10.1002/prot.10490) 12910454

[16] Oliveira L, Paiva AC, Vriend G (2002) Correlated mutation analyses on very large sequence families. Chembiochem 3: 1010–1017. (10.1002/1439-7633(20021004)3:10<1010::AID-CBIC1010>3.0.CO;2-T) 12362367

[17] Singer M, Vriend G, Bywater RP (2002) Prediction of protein residue contacts with a PDB-derived likelihood matrix. Protein Eng 15: 721–725. (10.1093/protein/15.9.721) 12456870

[18] Eyal E, Frenkel-Morgenstern M, Sobolev V, Pietrokovski S (2007) A pair-to-pair amino acids substitution matrix and its applications for protein structure prediction. Proteins 67: 142–153. (10.1002/prot.21223) 17243158

[19] Henikoff J, Henikoff S (1999) Blocks: non-redundant database of protein alignment blocks derived from multiple compilations. Bioinformatics 15: 471–479. (10.1093/bioinformatics/15.6.471) 10383472

[20] Rost B, Sander C (1993) Improved prediction of protein secondary structure by use of sequence profiles and neural networks. Proc Natl Acad Sci USA 90: 7558–7562. (10.1073/pnas.90.16.7558) 8356056 PMC47181

[21] De Moraes FR, Neshich IAP, Mazoni I, Yano IH, Pereira JGC, Salim JA, et al (2014) Improving predictions of protein-protein interfaces by combining amino acid-specific classifiers based on structural and physicochemical descriptors with their weighted neighbour averages. PLoS ONE 1 28 (10.1371/journal.pone.0087107) PMC3904977 24489849

[22] Mackay AL (1974) Generalised structural geometry. Acta Crystallographica A 30: 440–447. (10.1107/S0567739474000945)

[23] Crippen GM, Havel TF Distance Geometry and Molecular Conformation. Wiley New York (1988)

[24] Lund O, Hansen J, Brunak S, Bohr J (1996) Relationship between protein structure and geometrical constraints. Protein Science 5: 2217–2225. (10.1002/pro.5560051108) 8931140 PMC2143282

[25] De Groot BL, Van Aalten DMF, Scheek RM, Amadei A, Vriend G, Berendsen HJC (1997) Prediction of protein conformational freedom from distance constraints. Proteins 29:240–251. (10.1002/(SICI)1097-0134(199710)29:2<240::AID-PROT11>3.0.CO;2-O) 9329088

[26] Debe DA, Carlson MJ, Sadanobu J, Chan SI, Goddard WA III (1999) Protein fold determination from sparse distance constraints: The restrained generic protein direct Monte Carlo method. Journal of Physical Chemistry B 103: 3001–3008. (10.1021/jp983429+)

[27] Taylor WR, Hatrick K (1994) Compensating changes in protein multiple sequence alignments. Prot Engineering 7: 341–348. (10.1093/protein/7.3.341) 8177883

[28] Talavera D, Lovell SC, Whelan S (2015) Covariation is a poor measure of molecular coevolution. Molecular Biology and Evolution 5 4 pii: msv109 (10.1093/molbev/msv109) PMC4540965 25944916

[29] Kamisetty H, Ovchinnikov S, Baker D (2013) Assessing the utility of coevolution-based residue–residue contact predictions in a sequence-and structure-rich era. Proceedings National Academy of Science USA 110: 15674–15679. (10.1073/pnas.1314045110) PMC3785744 24009338

[30] Bywater RP (2016) A tensegrity model for protein structure. *In press 2016*.

[31] Morcos F, Pagnani A, Lunta B, Bertolino A, Marks DS, Sander C, et al (2011) Direct-coupling analysis of residue coevolution captures native contacts across many protein families. Proc. Natl. Acad. Sci. 108:E1293–E1301. (10.1073/pnas.1111471108) 22106262 PMC3241805

[32] Kolmogorov AN (1968) Three Approaches to the Quantitative Definition of Information. International Journal of Computer Mathematics 2: 157–168. (10.1080/00207166808803030)

